# The Challenge of Evaluating the Intensity of Short Actions in Soccer: A New Methodological Approach Using Percentage Acceleration

**DOI:** 10.1371/journal.pone.0166534

**Published:** 2016-11-15

**Authors:** Karin Sonderegger, Markus Tschopp, Wolfgang Taube

**Affiliations:** 1 Swiss Federal Institute of Sport Magglingen SFISM, Section for Elite Sport, Magglingen, Switzerland; 2 Department of Medicine, Movement and Sport Science, University of Fribourg, Fribourg, Switzerland; Universidad Europea de Madrid, SPAIN

## Abstract

**Purpose:**

There are several approaches to quantifying physical load in team sports using positional data. Distances in different speed zones are most commonly used. Recent studies have used acceleration data in addition in order to take short intense actions into account. However, the fact that acceleration decreases with increasing initial running speed is ignored and therefore introduces a bias. The aim of our study was to develop a new methodological approach that removes this bias. For this purpose, percentage acceleration was calculated as the ratio of the maximal acceleration of the action (a_max,action_) and the maximal voluntary acceleration (a_max_) that can be achieved for a particular initial running speed (percentage acceleration [%] = a_max,action_ / a_max_ * 100).

**Methods:**

To define a_max_, seventy-two highly trained junior male soccer players (17.1 ± 0.6 years) completed maximal sprints from standing and three different constant initial running speeds (v_init_; trotting: ~6.0 km·h^–1^; jogging: ~10.8 km·h^–1^; running: ~15.0 km·h^–1^).

**Results:**

The a_max_ was 6.01 ± 0.55 from a standing start, 4.33 ± 0.40 from trotting, 3.20 ± 0.49 from jogging and 2.29 ± 0.34 m·s^–2^ from running. The a_max_ correlated significantly with v_init_ (r = –0.98) and the linear regression equation of highly-trained junior soccer players was: a_max_ = –0.23 * v_init_ + 5.99.

**Conclusion:**

Using linear regression analysis, we propose to classify high-intensity actions as accelerations >75% of the a_max_, corresponding to acceleration values for our population of >4.51 initiated from standing, >3.25 from trotting, >2.40 from jogging, and >1.72 m·s^–2^ from running. The use of percentage acceleration avoids the bias of underestimating actions with high and overestimating actions with low initial running speed. Furthermore, percentage acceleration allows determining individual intensity thresholds that are specific for one population or one single player.

## Introduction

Soccer is an intermittent team sport characterized by large amounts of low-intensity actions interspersed with frequent bouts of high-intensity actions [1]. Time-motion analyses have been widely used to evaluate the intensity of actions and to quantify the physical load of trainings or games. The analyses typically employ locomotor categories such as walking, jogging, running, and sprinting, and are based on the distance covered or time spent within certain running speed thresholds. However, definitions of locomotor categories have varied considerably depending on the author and measurement system [2]. This precludes comparisons between studies [2, 3] and reveals the arbitrary character of locomotor categorization. Furthermore, recent publications have evidenced noticeable limitations in estimating work load using absolute speed thresholds because many high-intensity actions go undetected [2] due to the short distance covered despite having a high acceleration [4, 5]. Various authors have therefore emphasized the importance of monitoring acceleration and deceleration in intermittent team sports in order to attain a more realistic picture of physical load and therefore strengthen game analyses [4–6]. This approach seems particularly important due to the high metabolic demand associated with acceleration, even if the running speed is low or moderate [6].

In scientific literature, different absolute acceleration thresholds have been used when evaluating physical load: Akenhead et al. [7] defined three acceleration thresholds (low: 1–2 m·s^–2^, moderate: 2–3 m·s^–2^, high: >3 m·s^–2^) whereas Bradley et al. [8] differentiated between medium (2.5–4.0 m·s^–2^) and high (>4.0 m·s^–2^) accelerations. Aughey [9] analyzed the number of maximal accelerations by counting all accelerations higher than 2.78 m·s^–2^. Dwyer and Gabbett [2] established, in addition to the common sprint speed threshold, a new definition of sprint depending on the number and the amount of accelerations during games. Although the importance of considering acceleration is now widely accepted, a consistent classification does not yet exist. The abovementioned thresholds were chosen as appropriate arbitrary demarcations during match play [7] but were not developed systematically.

Acceleration is the change in speed over time ($a=\frac{\Delta v}{\Delta t}$). The largest increase in speed is at the beginning of the action, which then plateaus out with increasing running speed [10]. Therefore, the change in speed over time (= acceleration) decreases with increasing running speed, and maximal acceleration occurs at the beginning of the action. Thus, it can be assumed that maximal voluntary acceleration is lower when accelerations are initiated from low or moderate running speeds than from standstill. Therefore, commonly used absolute acceleration thresholds (e.g., > 3 m∙s^–2^) disregard the different acceleration capacities from different initial running speeds. As a consequence, absolute acceleration thresholds are hypothesized to underestimate actions with high initial running speed and overestimate actions with low initial running speed.

As soccer players often initiate actions from jogging or running (and not only from a stationary position) [5, 11], it is essential to know how maximal voluntary acceleration changes with different initial running speeds. Based on previous studies [12, 13], it can be assumed that accelerations initiated from different starting speeds cause differences in the neuromuscular preload, in the body inclines and therefore also in muscle group activation. Knowing the maximal voluntary acceleration of any given initial running speed allows to correctly interpret the level of acceleration during training sessions or games. However, the extent to which the maximal voluntary acceleration decreases when accelerations are initiated from different initial running speeds has, to our knowledge, not yet been examined and may be specific to a certain population.

The purpose of our study was to present a new methodological approach for evaluating the intensity of locomotor actions in soccer by taking into account the maximal acceleration capacity from different initial running speeds. Furthermore, the limitations associated with existing acceleration methods are demonstrated.

## Materials and Methods

### Participants

Seventy-two highly trained male junior soccer players (mean ± SD; age 17.1 ± 0.6 years; height 177.3 ± 5.9 cm; mass 69.9 ± 7.5 kg), belonging to seven different Swiss top-level teams (under 18 league), performed a specific sprint test. Before the start of the sprint test, each player completed a questionnaire about his health status. Players with impaired health were excluded from the test procedure. Participating players neither suffered from serious injuries or infections nor had trained intensely within the 48 h preceding testing. The study was approved by an independent Institutional Review Board of the Swiss Federal Institute of Sport, Magglingen, Switzerland. All tests were performed in accordance with the Declaration of Helsinki. Players received detailed verbal and written information about the study design before providing written informed consent. They were free to withdraw from the study at any time.

### Testing procedures

#### Sprint test

The test consisted of four maximally accelerated sprints over 50 m. This distance was chosen to achieve maximal running speed [14, 15]. The first sprint was performed from a standing start, while subsequent sprints were performed out of one of three constant initial running speeds (v_init,set_): trotting (6.0 km·h^–1^), jogging (10.8 km·h^–1^), and running (15.0 km·h^–1^). Higher initial running speeds were omitted because players were not able to consistently reproduce such high initial speeds with good accuracy. Recovery time between each sprint was 3 min. A paced runner who received short auditory signals through a headset ensured that players met the targeted v_init,set_ before acceleration. Markers on the pitch every 5 m indicated the distance covered between two auditory signals. Three players on each side of the paced runner adopted the target running speed. The players maintained the target running speed for 25–40 m and at an arbitrary time point, the paced runner blew a whistle, which signaled players to accelerate maximally and to run as fast as possible. As the players did not know exactly when the sprint would begin, anticipation and the early start of acceleration was precluded. Coaches provided verbal encouragement. All sprints were performed on the same artificial turf. Weather conditions were dry, windless and with temperatures of around 20°C. In the days preceding testing, all players completed a familiarization trial during a training session, consisting of a 25-min warm-up and the actual sprint test. Test-retest reliability was checked with two teams (22 players). The players completed the test two times with a break of one week.

#### Measurement system

The players’ positional data were measured using the Local Position Measurement (LPM) system (Inmotiotec GmbH, Regau, Austria). The LPM system recorded positional data by dividing 1000 Hz by the number of players. Although only six players performed their sprints at a time, the system always measured 36 players simultaneously for organizational reasons. Therefore, the temporal resolution was approximately 28 Hz. The LPM system produces highly accurate position and speed data [16, 17]; data transmission is described in Stelzer et al. [18].

### Data analysis

#### Initial running speed and maximal voluntary acceleration

**Fig 1:**
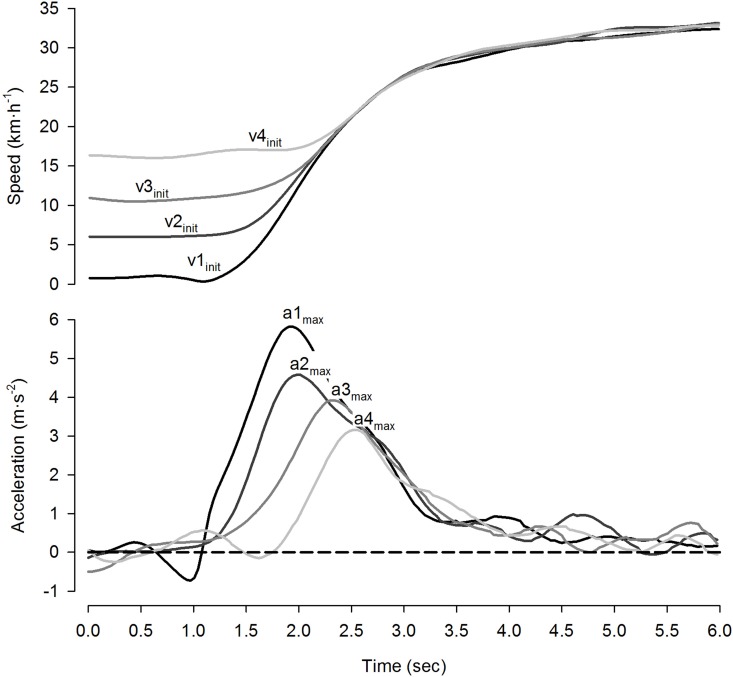
Speed (above) and acceleration (below) curves of the four sprints of one representative player. v1_init_ and a1_max_: effective initial running speed (v_init_) and maximal voluntary acceleration (a_max_), respectively, from standing; v2_init_ and a2_max_: v_init_ and a_max_, trotting; v3_init_ and a3_max_: v_init_ and a_max_, jogging; and v4_init_ and a4_max_: v_init_ and a_max_, running.

The initial running speed (v_init_), maximal running speed (v_max_) and maximal voluntary acceleration (a_max_) were extracted from the dataset. As the actual initial speeds varied slightly among players, the v_init_ of each player was used and not the v_init,set_. The v_init_ was defined as the running speed immediately before the start of acceleration. The v_max_ and a_max_ were determined as the highest speed and acceleration values during each of the four sprints. These three parameters were automatically extracted in Microsoft Excel (2007) and thereafter visually controlled with charts. The speed and acceleration curves of the four sprints of one representative player is illustrated in Fig 1.

#### Assessment of percentage acceleration

The new methodological approach evaluates the intensity of short locomotor actions during training sessions or games by using *percentage acceleration*. The percentage acceleration is the ratio of the maximal occurred acceleration of the action (a_max,action_) and the maximal voluntary acceleration (a_max_) that can be achieved for the particular v_init_:

$$Percentage\;acceleration\;[\%]=\frac{a_{max,\;action}}{a_{max}}*100$$ [1]

In the following parts of this paper, *percentage acceleration* is expressed for each initial running speed as the percentage of a_max_ from this particular running speed.

### Statistical analysis

Statistical analyses were conducted in SPSS Version 22.0 for Windows (SPSS Inc, Chicago, IL, USA). The one-way repeated measures analysis of variance (ANOVA) was used to examine mean differences in the a_max_ across the four different v_init_. Data were tested for normality using the Shapiro-Wilk test (all tests p > 0.05). The assumption of homogeneity of variance was verified with the Mauchly’s sphericity test. Statistical significance was set at p < 0.05. Effect sizes (ES) ± 95% confidence limits were calculated using standardized Cohen units [19] and categorized as trivial (<0.2), small (0.2–0.6), moderate (0.6–1.2), large (1.2–2.0), and very large (>2.0) [20]. The relationship between v_init_ and a_max_ was assessed using simple linear regression equation and Pearson correlation coefficient r. Data are presented as the means ± SD (±95% confidence limits). Test-retest reliability of a_max_ was reported with a paired *t* test and the magnitude of differences with ES statistics. The spreadsheet of Hopkins [21] was used to determine the typical error of measurement, and expressed as a coefficient of variation (CV).

## Results

Table 1 shows the effective initial running speed immediately before acceleration, the maximal voluntary acceleration and the maximal running speed of the highly-trained junior soccer players.

**Table 1:** Achieved maximal voluntary acceleration and maximal running speed out of four different initial movement speeds.

v_init_ (km·h^–1^)		a_max_ (m·s^–2^)	v_max_ (km·h^–1^)
Standing		6.01 ± 0.55 (5.88; 6.14)	31.3 ± 1.5 (30.8; 31.5)
Trotting	6.2 ± 0.8 (6.0; 6.4)	4.33 ± 0.40 (4.24; 4.43)	31.1 ± 1.4 (30.8; 31.4)
Jogging	11.4 ± 1.3 (11.1; 11.7)	3.20 ± 0.49 (3.08; 3.31)	30.7 ± 1.5 (30.4; 31.1)
Running	16.7 ± 1.2 (16.4; 17.0)	2.29 ± 0.34 (2.21; 2.37)	30.7 ± 1.5 (30.3; 31.0)

Values are expressed as mean ± Standard deviation (95% CL). v_init_ = initial running speed before acceleration started; a_max =_ maximal voluntary acceleration; v_max_ = maximal running speed.

Effects of v_init_ on a_max_ were statistically highly significant (*F*_3, 213_ = 1287, *P* < .0001). ES for differences of a_max_ were –3.5 (–4.0 to –3.0) from standing to trotting, –2.5 (–3.0 to –2.1) from trotting to jogging, and –2.2 (–2.6 to –1.8) from jogging to running.

**Fig 2:**
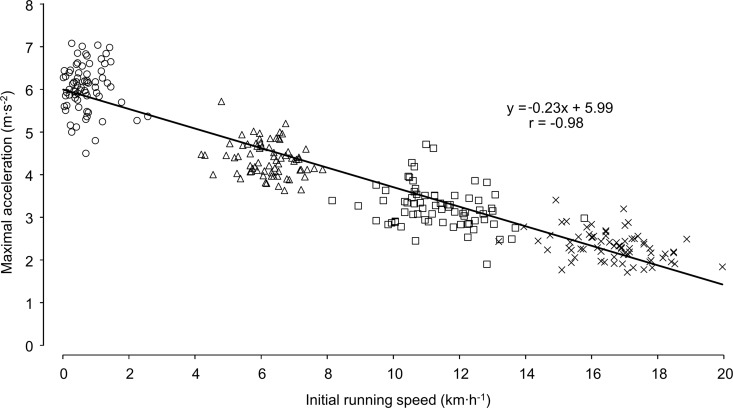
Maximal voluntary accelerations for standing (circles), trotting (triangles), jogging (squares), and running (crosses) of 72 highly trained male soccer players (under 18 league). Simple linear regression line with regression equation and Pearson’s correlation coefficient r.

The a_max_ correlated significantly with the v_init_ (r = –0.98, 95% CL: –0.97 to –0.98) and decreased linearly (Fig 2). In this sample of highly trained junior male soccer players, the group-specific decrease of a_max_ with increasing initial running speed is characterized with the following linear regression Eq (2):

$$a_{max}=-0.23\;(-0.22\;to-0.24)*v_{init}+5.99\;(5.87\;to\;6.11),$$ [2]

where a_max_ is in m·s^–2^ and v_init_ in km·h^–1^. Values in parentheses are 95% CL.

**Fig 3:**
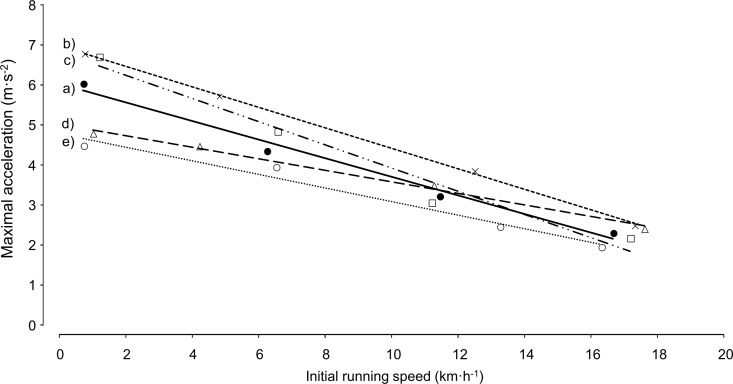
Individual linear regression lines of a_max_ depending on v_init_. (a) Mean of all soccer players (n = 72) (black circles); (b) player with high a_max_ at all v_init_ (cross); (c) player with high decrease in a_max_ with increasing v_init_ (square); (d) player with low decrease in a_max_ with increasing v_init_ (triangle); (e) player with low a_max_ at all v_init_ (white circle).

The standard deviation (SD) of the slope of ±0.03 and the SD of the intercept of ±0.51 indicates the variation of the individual linear regression line. Fig 3 shows exemplary 4 representative individual gradients for a_max_ depending on v_init_.

Pairwise analysis of reliability of a_max_ revealed no significant differences (all p > 0.05) and trivial to small effects (ES < 0.4) between test-retest (from 0 km·h^–1^: a_max_ = 5.8 ± 0.4 vs. 5.6 ± 0.6 m·s^–2^; from 6 km·h^–1^: a_max_ = 4.5 ± 0.4 vs. 4.4 ± 0.4 m·s^–2^; from 10.8 km·h^–1^: a_max_ = 3.5 ± 0.3 vs. 3.4 ± 0.3 m·s^–2^; from 15 km·h^–1^: a_max_ = 2.5 ± 0.4 vs. 2.5 ± 0.4 m·s^–2^). The coefficient of variation of a_max_ was 6.7% (5.4–9.2) from 0 km·h^–1^, 5.4% (4.3–7.3) from 6 km·h^–1^, 6.1% (4.9–8.3) from 10.8 km·h^–1^, and 10.9% (8.6–14.9) from 15 km·h^–1^.

## Discussion

Our results show a linear decrease in the maximal voluntary acceleration when sprints are initiated from increasing initial running speed. This reveals the importance of considering the running speed prior to acceleration when acceleration measurements are used to evaluate the intensity of short locomotor actions. Nevertheless, most of the actual studies that classified motor actions based on acceleration measures disregarded initial running speeds and used absolute acceleration thresholds. Therefore, we propose a new methodological approach relying on the quantification of locomotor actions according to percentage acceleration instead of absolute acceleration values.

By using absolute values, an action with an acceleration of 3 m·s^–2^ is often classified as an action with high acceleration [7, 22]. However, our results show that an acceleration of 3 m·s^–2^ represents only 50% of the maximal voluntary acceleration when initiated from a standing position. In contrast, from an initial running speed of 15 km·h^–1^, only a few highly trained young soccer players could reach an acceleration of 3 m·s^–2^. Therefore, the advantage of our new approach is that accelerations initiated from standing or low-speed running are not overestimated and accelerations from high-speed running are not underestimated, as is the case with commonly used absolute acceleration thresholds.

Although the results reported by Varley and Aughey [5] indicated the problem with absolute acceleration thresholds, the authors did not explicitly address this issue. They showed that nearly half of all actions with “maximal” accelerations > 2.78 m·s^–2^ were initiated from < 3.6 km·h^–1^ and only 8% of these “maximal” accelerations were realized when the initial running speed was over 10.8 km·h^–1^. The authors interpreted this finding in a rather misleading way and concluded that actions with maximal acceleration very rarely occur with initial running speeds exceeding 10.8 km·h^–1^. However, based on our results, which illustrate a pronounced decrease in the maximal voluntary acceleration with increasing initial running speed, it seems more likely that even if the players accelerated maximally, they would not able to exceed the absolute value of 2.78 m·s^–2^ if the initial running speed was over 10.8 km·h^–1^.

Dwyer and Gabbett [2] classified sprints based on both speed and acceleration. In four different speed zones, actions with the highest 5% of acceleration were also defined as sprints. With their definition of sprint, even *higher* acceleration out of jogging or running was necessary for an action being qualified as a sprint than from an initial status of standing or walking. Therefore, the existing problem caused by the absolute acceleration threshold was even exaggerated by their approach and probably reinforced by two issues: first, the dataset from their study was generated during games and the maximal voluntary acceleration was not known. Second, a large number of movements were initiated from low initial speeds or even from a stationary position. Thus, when taking the 5% of actions with the highest acceleration, this might include many submaximal trials, consequently underestimating the capacity to accelerate maximally from this low speed zone. It therefore seems vital to measure the maximal voluntary acceleration during an isolated sprint test (for a given initial speed) and not during a game in order to ensure that the acceleration was maximal and thus, to avoid misclassification. Another reason for an isolated sprint test is that factors such as age, performance level, and gender can be taken into account for the calculation of percentage acceleration.

Percentage acceleration (Eq (1)) can be calculated by the ratio of the maximal occurred acceleration of the action (a_max,action_) and the maximal voluntary acceleration (a_max_) for this specific initial running speed (linear regression Eq (2)). In order to classify intensity of actions based on percentage acceleration, we propose four different intensity zones: (1) actions with high percentage accelerations (acceleration >75% of the a_max_), (2) actions with moderate percentage accelerations (acceleration >50% of the a_max_), (3) actions with low percentage accelerations (acceleration >25% the of a_max_), and (4) actions with very low percentage accelerations (acceleration ≤25% of the a_max_). Therefore, an action with an acceleration of >4.51 from standing, >3.25 from trotting, >2.40 from jogging, and >1.72 m·s^–2^ from running corresponds to an action with a high percentage acceleration. The physical load of a training session or soccer game can be estimated by quantifying the number of actions with high, moderate, low and very low percentage accelerations.

A group-specific linear regression equation (Eq 2) has been developed in this study for highly trained soccer players under 18 years of age. For another population, an adapted regression equation may be more appropriate. Furthermore, it should be noted that subjects varied in their level of maximal voluntary acceleration, and therefore, as shown in Fig 3, the slope and intercept of the regression line obtained for the group does not represent the individual subject perfectly. Thus, it is obvious that individual regression equations for calculating percentage acceleration, rather than group-specific equations, give a better indication of the exploitation of the individual acceleration potential. On the other hand, the fact that direct comparisons between players are possible when using a group-specific regression equation may be considered as advantage of this type of approach.

It should be noted that with Eq 2, the calculated maximal running speed is 26 km·h^–1^, which is markedly lower than the effective measured maximal running speed (about 31 km·h^–1^; which corresponds to previously reported running speeds [15]). The reason for this is that the relationship between a_max_ and v_init_ is probably not perfectly linear over the entire range of speed and our measurements were performed with initial speeds of only up to 15 km·h^–1^.

Although this new methodological approach certainly improves the quantification of short intense locomotor actions, vertical jumps, rotations, and decelerations are still disregarded. Furthermore, the accuracy of this approach depends not only on the athletes’ motivation when determining maximal voluntary acceleration (i.e., they have to perform the test with maximum effort) but also on the accuracy of the measurement system to assess the subjects’ changes of position. Stevens et al. [23] reported that the measurement errors of the LPM system are greater when assessing accelerations than when assessing distance or speed. This finding is not surprising as time-motion analysis systems record position data per time interval and acceleration is calculated with the second derivation of the position displacement data. Nevertheless, the LPM system shows a lower coefficient of variation for maximal acceleration compared to the global positioning system (GPS) or other position measurement systems [24]. Thus, although the newly developed methodological approach presented in this paper was developed using the LPM system, it can be transferred to other position-detecting systems. However, the limitations of the measurement system should always be kept in mind.

## Conclusions

This study presents a new methodological approach to evaluate the intensity of short actions in soccer by considering the fact that the maximum voluntary acceleration decreases with increasing initial running speed. The new methodological approach introduces percentage acceleration, which is calculated as the ratio of the maximal acceleration of the action and the maximal voluntary acceleration that can be achieved for a particular initial running speed. Using percentage instead of absolute acceleration thresholds helps avoid the bias of underestimation of actions with high initial running speed and overestimation of actions with low initial running speed.

When actions are classified in high, moderate, low and very low percentage accelerations, the physical load of training sessions or games can be estimated by quantifying the number of actions in these four intensity zones.

To guarantee an accurate interpretation of the percentage acceleration, group-specific or even individual linear regression equations should be considered.
